# Drivers of deforestation in the basin of the Usumacinta River: Inference on process from pattern analysis using generalised additive models

**DOI:** 10.1371/journal.pone.0222908

**Published:** 2019-09-25

**Authors:** Raúl Abel Vaca, Duncan John Golicher, Rocío Rodiles-Hernández, Miguel Ángel Castillo-Santiago, Marylin Bejarano, Darío Alejandro Navarrete-Gutiérrez

**Affiliations:** 1 CONACYT—Consorcio de Investigación, Innovación y Desarrollo para las Zonas Áridas (CIIDZA), El Colegio de San Luis (COLSAN), Fraccionamiento Colinas del Parque, San Luis Potosi, S.L.P., México; 2 Department of Life and Environmental Sciences, Bournemouth University, Poole, Dorset, United Kingdom; 3 Laboratorio de Análisis de Información Geográfica y Estadística, El Colegio de la Frontera Sur, San Cristóbal de Las Casas, Chiapas, México; 4 Pronatura Sur A.C., San Cristóbal de Las Casas, Chiapas, México; Indiana State University, UNITED STATES

## Abstract

Quantifying patterns of deforestation and linking these patterns to potentially influencing variables is a key component of modelling and projecting land use change. Statistical methods based on null hypothesis testing are only partially successful for interpreting deforestation in the context of the processes that have led to their formation. Simplifications of cause-consequence relationships that are difficult to support empirically may influence environment and development policies because they suggest simple solutions to complex problems. Deforestation is a complex process driven by multiple proximate and underlying factors and a range of scales. In this study we use a multivariate statistical analysis to provide contextual explanation for deforestation in the Usumacinta River Basin based on partial pattern matching. Our approach avoided testing trivial null hypotheses of lack of association and investigated the strength and form of the response to drivers. As not all factors involved in deforestation are easily mapped as GIS layers, analytical challenges arise due to lack of a one to one correspondence between mappable attributes and drivers. We avoided testing simple statistical hypotheses such as the detectability of a significant linear relationship between deforestation and proximity to roads or water. We developed a series of informative generalised additive models based on combinations of layers that corresponded to hypotheses regarding processes. The importance of the variables representing accessibility was emphasised by the analysis. We provide evidence that land tenure is a critical factor in shaping the decision to deforest and that direct beam insolation has an effect associated with fire frequency and intensity. The effect of winter insolation was found to have many applied implications for land management. The methodology was useful for interpreting the relative importance of sets of variables representing drivers of deforestation. It was an informative approach, thus allowing the construction of a comprehensive understanding of its causes.

## Introduction

The process of deforestation rarely if ever takes place in a haphazard random fashion. Areas are chosen for conversion to other land uses on the basis on a set of favorable, facilitating characteristics. Access to roads, for example, may be an important consideration in a decision to deforest a particular patch of forest. Similarly, lower elevations and gentle slopes may be preferred for agriculture, owing to potentially higher land preparation and management costs in rugged terrain. Patterns emerge over a landscape that are the result of these choices [1–2]. Forest loss does also arise as a result of unpredictable processes, such as fire. Fires are determined by complex interactions between climate, land use, vegetation attributes, and the pattern of ignition [3–6]. The behavior of grazing animals and the role of uncontrolled trampling and browsing in preventing regeneration can be unpredictable, although grazing is also linked to choices made by people [7–9]. The complexity is compounded by historical contingency. Optimal decisions regarding land use are constrained by social and political factors [10–12]. Land tenure plays a critical role in shaping the decision to deforest [13–15]. The combination of all these factors shapes the deforested landscapes we observe [16–17]. These landscapes may have a clear spatial pattern, but interpreting this pattern in the context of the processes that have led to its formation may be challenging [16,18–19]. The intention behind statistical analysis of this spatial pattern is to tease apart the elements of complexity and guide explanations based on knowledge of underlying processes. This knowledge can then be used to predict future deforestation or can be fed back into the policy domain in order to shape the decision making process.

Conventionally, deforestation has been analyzed by using traditional statistical methods that are only partially successful for interpreting its pattern in the context of responsible processes [1,20–21]. Common understanding of the causes of deforestation is dominated by simplifications which, in turn, underlie many environment-development policies [1,22]. Traditional methods suffer from a set of weaknesses that arise when observational data on spatially explicit phenomena have not arisen as the result of planned experimentation, for example: (1) correlation cannot be taken as a direct indication of causation; (2) spatial autocorrelation can exaggerate relationships due to lack of independence; (3) statistical models that can be selected for prediction may have little explanatory value; (4) relationships can be non linear; (5) variables that cannot be mapped onto space could be critical factors. Theoretical and applied ecologists are becoming increasingly dissatisfied with the traditional testing-based aspects of statistics [23]. A large body of statistical literature has shown the testing of null hypotheses to have relatively little utility, in spite of their very widespread use [24–27]. The problem with the statistical null hypothesis testing approach is that it is relatively uninformative [23,28].

The statistical analysis of deforestation in the basin of the Usumacinta River adopted a methodology that was explicitly designed to address these issues, allowing the construction of a comprehensive understanding of the causes of deforestation that can supports research and inform decision making. Spatially explicit layers of drivers were chosen for analysis that could be linked to known process. Statistical correlation was not taken as indication of causation. Instead the strength and functional form of relationships was looked at using a methodology that partialed out effects in the presence of competing drivers. Non linear models were fitted that allowed the shape of relationships to be visualised, described and explained. A critical discussion was included of further historical factors responsible for deforestation. The aim of the study is to determine the relative importance of a series of physical and socio political factors that have determined the pattern of deforestation in the basin of the Usumacinta River, a region identified as a hotspot of tropical and Mesoamerican biodiversity and one of the Mexican most biologically rich areas [29]. We had two specific objectives when designing our analytical framework: (1) to separate factors influencing native vegetation cover and produce a series of informative raster coverages that mapped these factors onto space; and (2) to analyses the relative importance of these drivers by comparing spatial patterns of drivers with the observed pattern of recent deforestation (2000–2014) over different landscapes within the Usumacinta River Basin.

### Study area

**Fig 1:**
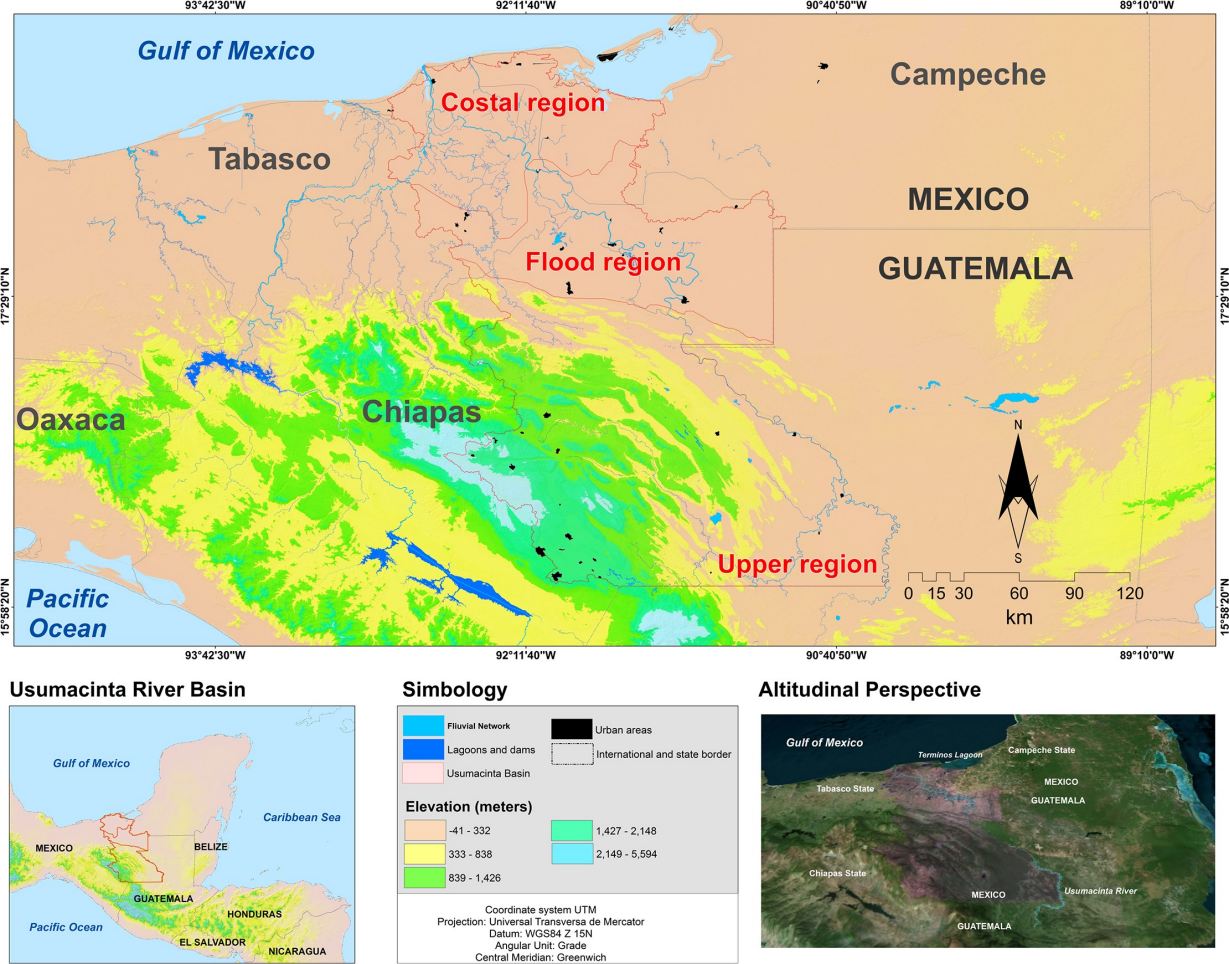
Study area. Basin of the Usumacinta River in Mexico. The study area consists of the complete area of the Usumacinta River Basin located in Mexican territory. While significant portions of the basin are located in Guatemala, the Mexican portion (study area) includes a marked physiographic and environmental gradient that divides the region into upper, middle, and lower basins.

The study area consists of the complete area of the Usumacinta River Basin located in Mexican territory (Fig 1). It is located between 16°04’ and 18°41’ N latitude and 90°19’ and 93°00’ W longitude, and its surface area totals 41 180 km^2^. The basin of the Usumacinta is shared by Guatemala and Mexico, and drains one of the most biologically diverse regions in the world as well as one of the largest remaining contiguous tropical forest areas north of the Amazon [29]. The Usumacinta River also flows through the most economically and politically marginalized regions of both Guatemala and Mexico [29]. The part of the basin that lies within Mexican territory ranges in altitude from zero to 2600 m.a.s.l. and can be divided into three different parts, which differ in physiography, hydrography, and ecological character: upper basin (200–2600 m.a.s.l.) (25 368.1 km^2^), middle basin or floodplain (50–200 m.a.s.l.) (7330.2 km^2^) and lower basin or coastal plain (0–50 m.a.s.l.) (8481.7 km^2^).

Rainfall in the region is the highest in the country. The annual average is 2143 mm. In some parts of the upper basin rainfall exceeds 4000 mm per year and may reach 5000 mm. In the northern coastal area, rainfall averages 2093 mm per year and may reach 2750 mm. It rains almost all year; spring is the only relatively dry season. The annual mean temperature is 24°C [30]. Most of the study area was probably originally covered with tropical vegetation [31–32]; however, recent research [33] has shown that the extent of native vegetation in some parts of the study region has been reduced to less than 30% of its original area. The greatest loss of forest area occurred in the period from 1930s to 1980s with the needs for agricultural and livestock expansion. The main types of vegetation include pine-oak forest at higher altitude, montane cloud forest and tall evergreen forest at mid-altitude, and medium semi-deciduous forest, shrubland and hydrophytic vegetation at lower altitude [34].

Most of the territory of the Usumacinta River Basin has a predominantly agricultural economy. Historical deforestation has been driven mainly by cattle grazing and agriculture expansion, affecting most of the lowland areas of Tabasco [35–36]. Tabasco holds the highest population density (80.5 persons/km^2^) as compared with the rest of the states of Southern Mexico. Many municipalities in Tabasco are associated with a high population density since the beginning of the XX century, when a railway and highway were constructed connecting the major cities in the region [37]. Increased communication promoted the development of activities such as timber trading, sugar factories, tobacco plantations, and cattle ranching, which have had a marked impact on the forests [37]. In the last decades, deforestation has continued to affect the study region, but the rate of change has slowed and been displaced into some new areas including the mid-altitude forests of the Lacandon region [33]. In these frontier parts of the basin governments have encouraged immigration, and policies that promote livestock farming have been among the principle causes motivating deforestation. In addition, land tenure is commonly uncertain or contested, and there is ineffective enforcement against incursions into parks or other protected areas, thus immigration has often resulted in deforestation of protected areas or other inappropriate lands [38–39]. Annual cropping, mostly for maize and beans, is the land use whose expansion may have contributed most to historical deforestation of upland forests [33].

Human settlement and urbanization have also contributed to permanent deforestation and intensification in land use [40].Oil and petrochemical development propelled rapid growth of urban centres in the coastal region (i.e. lower basin) and shifted the proportion of rural dwellers to urban population. Villahermosa, the capital city of Tabasco, is located in the lower basin and is the largest urban centre in the study region, with a population of 353 577 inhabitants. Oil has been exploited in the area since the 1930's. At present, exploration and development is continuing in more remote parts of the basin. Some of the greatest concerns of petroleum exploitation are for the indirect effects of building roads and bringing workers into forested areas [38].

In most municipalities of the study region population density is below 50 people per km^2^. Land tenure is unequally concentrated. Chronic rural deprivation is a feature of the study region. The Usumacinta river basin offers many potentialities for development whereas the local inhabitants are among the poorest in Central America [39]. A large proportion of those living in conditions of high marginality and poverty are found in rural agricultural areas, and they are predominantly of indigenous ethnicity and subsistence–farmers [39]. The presence and maintenance of extreme poverty in these rural areas is rooted in the historical context of unequal land distribution, on the quality of land distributed by the government, on the small parcels of subsistence farmers which are often times not entirely arable, and on the overall unfavorable state of rural infrastructure needed to facilitate the distribution of products [41]. In the study region, the most profitable land is retained into private properties within which extensive agricultural and cattle raising activities are developed by high and middle-income landowners.

## Materials and methods

### Data sources

We used a land cover and use classification map for the study area and the year 2015, which was performed following a supervised classification of LANDSAT 8 OLI imagery. An accuracy assessment of the classification results suggested a Kappa index of agreement of 0.85, and an overall agreement of 90.2 percent. For the year 2000, we used a land cover classification of Landsat Enhanced Thematic Mapper Plus (ETM+) imagery. The Kappa index of agreement was 0.88, and overall agreement was 92.2 percent. The pixel size of the images was 30 m. Classification maps were overlaid in order to produce a map of deforestation that represents changes in native vegetation cover. Our analysis was based on a minimal set of informative classes: natural cover and deforestation coded 1 and 0, respectively. The 'natural cover' class included forest (old growth forest as well as secondary and degraded forest), shrubland, wetland and regeneration. The 'deforestation' class depicted areas that have been cleared of native vegetation during the period 2000–2014.

### Physical and socio political factors influencing forest cover

We formed two sets of hypotheses that we could test using generalised additive models: (1) hypotheses concerning factors that affect vegetation directly (i.e. climate, soils, natural disturbance—fire, wind -, geomorphology, hydroperiod); and (2) hypotheses concerning factors affecting decisions taken to deforest (i.e. accessibility, pressure, opportunity cost—value of alternative land use above forest -). The first set of hypotheses fall largely into the category of facilitating elements, while the second can be considered as underlying and proximate drivers [14]. Drivers were derived from a digital elevation model, piezometric information, population census data, and vector layers representing roads, streams and soil types, using the open source geographical information system GRASS GIS [42]. Table 1 provides details of the layers used in the analysis and the GRASS functions used in their calculation.

**Table 1:** Layers used in the analysis. Raster coverages representing drivers, and the layers and GRASS functions used in their calculation.

Input layers	GRASS function	Output
Digital elevation model (INEGI 30m resolution)	r.slope.aspect	**slope, aspect**
Annual rainfall (derived from climatic stations, DEM, and universal kriging)		**AnnualPrec**
Road network (INEGI 1:5000)	r.buffer input = roads output = roadsbuffer distances = 100,200,500,1000,5000,10000,50000 units = meters	**roadsbuffer** Used for calculating cost of access.
Census data for the year 2010 (INEGI) Slope (derived from DEM) Road network (INEGI 1:5000)	v.extract input = census2010 type = point output = cities where = "TotalPopulation>10000" r.mapcalc 'cost = 1 + (slope/10) + (roadsbuffer/2)' r.cost input = cost output = Access10000 start_points = cities max_cost = 0	**Access10000** Relative cost surface derived from distance from cities with more than 10000 inhabitants. Travel by road is given lowest weight. Slope and distance from roads are considered as contributing to the cost of access.
Census data for the year 2010 (INEGI) Slope (derived from DEM) Road network (INEGI 1:5000)	v.extract input = census2010 type = point output = towns where = "TotalPopulation>100" r.mapcalc 'cost = 1 + (slope/10) + (roadsbuffer/2)' r.cost input = cost output = Access100 start_points = towns max_cost = 0	**Access100** Relative cost surface derived from distance from villages with more than 100 inhabitants. Travel by road is given lowest weight. Slope and distance from roads are considered as contributing to the cost of access.
Census data for the year 2010 (INEGI) Land tenure (*ejidos* properties with resident rural communities, and private properties)	v.surf.icw input = census2010 column = TotalPopulation output = PopDens cost_map = LandTenure	**PopDens** Total population at the pixel level derived by cost weighting interpolation from irregularly spaced vector data points. Solid barriers (pattern of land tenure) are taken into account
Digital elevation model (INEGI 30m resolution) Slope (derived from DEM) Aspect (derived from DEM)	r.topidx	**Topidx** Topological index that captures geomorphology as a combination of leading slope length and inclination. High values represent areas at the base of slopes where soil and moisture accumulate
Digital elevation model (INEGI) Aspect and Slope (derived from DEM) Day of year (1 January) Latitude (Single value used) Links turbidity coefficient (estimated)	r.sun elevin = DEM aspin = Aspect aspect = 270 slopein = Slope slope = 0.0 lin = 3.0 alb = 0.2 lat = 16 beam_rad = Beam day = 1 step = 0.5 dist = 1.0 numpartitions = 1	**Beam** Layer representing direct beam radiation in Wh/day/m^2^
Piezometric data	v.surf.idw input = PiezometricData column = PiezometricLevel output = Hydroperiod	**Hydroperiod** Piezometric level derived by surface interpolation from irregularly spaced vector data points and Inverse Distance Squared Weighting

*Elevation* Deforestation is hypothesised to be less likely on high elevations and rugged terrains, since the terrain may increase site preparation costs and reduce returns to land cleared for agriculture. Another element associated with elevation is the effect of elevation on air temperature which determines forest type. The cooler conditions at elevations above 1000 m tend to favour the pines and oaks over the more diverse tropical forests. The composition of the forest will influence decisions in diverse ways. Pine forest at higher altitude may be more prone to deforestation due to the value of pines as timber. Conversely valuable forest may be considered a resource and managed. High air temperatures, on the other hand, increase evapotranspiration leading to an increase in the hydric stress experienced by plants during the dry months therefore preventing regeneration and favouring permanent deforestation. Hypotheses concerning the role of elevation in driving deforestation can be evaluated using a digital elevation model.

*Variations in rainfall* Annual precipitation is a factor that may influence the probability of deforestation as a result of multiple associated factors. There is considerable variability in rainfall over the study area. Moister conditions can favour rapid regeneration of forest after disturbance and reduce the frequency of fires. Another element associated with precipitation is the influence of precipitation on agricultural activities and the choice of crop.

*Natural disturbances* Vegetation loss may arise as a result of unpredictable processes such as fire. However fire is linked to human behaviors and physical process. Dry season insolation, for example, is thought to have an important role as a factor associated both with fire frequency and intensity and hydric stress. Therefore hypotheses concerning the effect of fires can be partially evaluated using direct beam insolation as a proxy. It was derived from the digital elevation model and for a clear day in the middle of the dry season (January 1).

*Soils* There are a series of difficulties associated with the use of available soil coverages. Coarse resolution soil maps in vector form are known to have a very weak and very ambiguous relationship with measured soil fertility on the ground. Maps are outdated and have not been validated recently. Soil maps have been derived at least in part as a result of the interpretation of geomorphology in an undocumented way. Polygons can coincide with rivers and roads. This confounds interpretation. We therefore decided that the coarse scale of available soil maps rendered them unsuitable as tools for forming testable hypotheses in this particular context.

*Geomorphology* Hypotheses concerning the effect of soils can be evaluated using relevant and interpretable variables that summarise different aspects of geomorphology. Specifically, geomorphology was represented by a topological index which was calculated as a function of slope length and steepness. Inclination, length and orientation of the slopes influences soil properties such as depth and organic matter content by affecting erosion and deposition of materials. As a result of its derivation the topological index represents areas where water and soil accumulate. The topological index is therefore closely correlated with distance from rivers and streams making the inclusion of secondary variables derived from the vector representation of water courses redundant.

*Hydroperiod* The hydroperiod defines the seasonal pattern of surface and subsurface water levels. This is conditioned by topography, climate, the seasonal patterns of water inlet and outlet, and tides. The conditions imposed by the hydroperiod may be very important for agriculture in floodplain areas because they affect several factors such as soil anaerobiosis and water accumulation in the surface, all of which influence soil suitability for agricultural activities. Hypotheses concerning the effect of the hydroperiod can be evaluated using the piezometric levels along the basin as an indicator of the hydrological behavior. The static or piezometric level refers to the depth to which the water of an aquifer is found. A continuous raster coverage was calculated using interpolation analysis.

*Slope* Slope is a factor that may influence the probability of deforestation as a result of multiple associated factors. Steep slopes can impose severe limitations to cropland or pastures which may arise from severe susceptibility to water or wind erosion, low moisture-holding capacity and frequent overflows accompanied by severe crop damage. Steeper slopes may also be linked both to increased difficulty to deforest and decreased capability of controlling burning (which is a common practice in slash-and-burn agriculture).

*Socio economic factors* can be summarised as “pressure”. Hypotheses concerning the effect of land pressure (producer’s behaviour) were evaluated using population density and cost of access:

*Population density* Local population in rural areas tends to locally produce goods for subsistence. Because most of their livelihoods are in subsistence farming, rural population growth is thought to propel continued expansion of agricultural area through the conversion of forests and wetlands. Also urban centers and urban populations may determine deforestation. In Southern Mexico the growth of many cities has been a consequence of immigration from the rural hinterland, and the use of nearby areas may have intensified due to the extraction of elements such as charcoal, fuelwood, timber, food, and other goods for the urban market. Hypotheses concerning the effect of population pressure on available land can be evaluated using total population at the pixel level. Patterns of land tenure were included as solid barriers for the calculation of total population at the pixel level.

*Local and regional relative accessibility* The selection of areas for agricultural expansion may be especially constrained or influenced by cost of access to available land. Therefore, cost of access to available land from rural settlements is thought to have an important role as a factor associated with land pressure and probability of deforestation. Urban centers may also influence demand for agricultural outputs, market availability, and other factors that impact input and output prices, farm size and ultimately smallholder behavior (i.e. inducing a supply response from farmers) and agricultural intensification. Hypotheses concerning producer’s behaviour can be evaluated using two different models: regional relative accessibility or accessibility to regional markets (cost of access from towns of over 10 000 inhabitants) and local relative accessibility (cost of access from villages of over 100 inhabitants). Accessibility can be measured as “cost surface”. Point of origin may be urban areas or rural communities. Distance from road network may be important and off road cost of access may be some function of slope. Therefore, distances from roads and distance from population centres were combined to produce two accessibility indices based on cost of access from towns of over 10000 inhabitants or communities of 100 inhabitants. Slope was taken into account in the calculation of cost of access.

### Statistical analysis

We divided the study region into upper, middle and lower basin in order to analyses and compare the relative importance of drivers in each of these contrasting landscapes. We conducted most of our analysis using the R statistical language [43] and the open source geographical information system GRASS GIS [42]. Raster coverages representing the response variables and drivers were imported into R from GRASS.

A sample of 2000 (for the middle and lower basins) and 3000 (for the upper basin) randomly selected pixels were used for model building. A series of generalised additive models (GAMs) of the binomial family were fitted using the R package MGCV [44]. Generalised additive models of the binomial family are related to logistic regression. They allow non linear responses to be modelled. The analysis aimed to assess the relative importance of each driver in association with the effects of other potential drivers of deforestation. The approach involves building multiple statistical models. Although the best fitting model might be preferred using purely statistical criteria, other models that represent processes can be considered providing they do not reduce the total fit by a large amount [23–24]. Information criteria is often used as an automated form of mediating between models, but should not be used alone if spatial autocorrelation can occur or if sample sizes are large [45]. The statistical significance of each term in a joint additive model can be calculated by comparing the chi-squared statistic to the chi-squared distribution with k degrees of freedom. However this *p* value is critically dependent on the number of pixels included in the sample drawn from the raster coverage. The value is arbitrary, and can be increased by taking a larger sample [28]. Thus statistical significance as a test of a null hypothesis if no association is not directly interpretable [26]. *P* values can be used as a relative measure of association; however, explained deviance is an interpretable measure of the strength of the association. The deviance explained by each variable modelled separately can be contrasted with the total deviance explained by a model that includes all the variables. Thus the proportion of the explainable deviance attributable to each driver alone can be extracted and compared against the total explainable deviance. This provides a measure of the comparative strengths of the potentially non linear association between the drivers and the proportion of forested pixels that does not depend on the arbitrary size of the sample, nor the order in which the drivers are included in the model. Also, the partial deviance explained for each variable in the complete model can be calculated by dropping a term from the model that includes all the drivers. Thus the relative importance of each variable in the presence of other variables can also be obtained.

The fit of a series of models of varying complexity was evaluated using information criteria (AIC). However because of the arbitrarily large sample size and the inflation of the denominator degrees of freedom due to autocorrelative effects this tends to result in the most complex models always being selected, which is apt for spatial prediction, but less adequate for explanation. Thus a model including all variables had the greatest predictive power. The subsequent explanatory analysis in terms of direct drivers emphasised the deviance explained and the form and shape of the relationships. As GAM models are based on spline functions it is essential to visualise the functional forms of the relationships graphically.

Autocorrelation in the response and explanatory variables was investigated by fitting variograms to the sampled points using the R package gstat [46]. The range at which autocorrelation becomes negligible varied from 10 km in the case of the response variable to 20 km in the case of slope and accessibility. Autocorrelation in both the drivers and the response variable can thus be regarded as an intrinsic part of the pattern being analysed and could not be entirely removed through sampling design. However direct beam radiation had a range of autocorrelation below the median distance between points (<1km) thus each data point could be assumed to be independent for this particular driver, giving it the highest direct explanatory value. Rainfall had the greatest autocorrelation giving it the lowest explanatory power.

In order to complement the GAM analysis and provide a direct interpretation of the strength of the drivers recursive partitioning was also used as implemented in the R package rpart [47]. Recursive partitioning models allow interactive effects between variables to be investigated.

## Results

A model which included elevation and annual precipitation explained the largest proportion of the deviance in all the cases: upper (26.8%), middle (11.4%) and lower (22.1%) basins. However, elevation was found to be highly correlated with slope and annual precipitation; and annual precipitation was found to be highly correlated with hydroperiod. Elevation and annual precipitation were therefore excluded in the explanatory phase of the analysis that concentrated on potential drivers.

Local relative accessibility and slope in the upper basin become the variables most strongly associated with the probability that a pixel remained forested (Table 2). Both variables explained the greatest proportion of the total deviance as well as a relatively high proportion of the deviance in the presence of other variables. Accessibility to regional markets and population density were the second most important variables both when taken alone and within a multivariate model. Direct beam radiation, topological index and hydroperiod explained a negligible part of the deviance in the multivariate model. A model which includes slope, local relative accessibility, accessibility to regional markets and population density (Table 3) explained the major part (24.4%) of the total deviance explained by the GAM model that includes all variables (25.1%).

**Table 2:** Results from generalised additive models. Percentage of the deviance explained by models built using single variables separately and partial deviances in the presence of all competing variables. The last column shows the deviance explained by each univariate model expressed as a percentage of the total deviance explained by the most complex multivariate model.

Region	Variable	Deviance	Partial deviance	Deviance proportion
Upper basin	Access100	10.3	10.2	41.0
	Access10000	4.8	1.2	19.1
	Beam	4.6	0.2	18.3
	Slope	10.3	3.8	41.0
	PopDens	3.9	2.4	15.5
	TopIdx	0.2	0.3	0.8
	Hydroperid	2.0	0.2	8.0
Middle basin	Access100	4.8	2.1	53.9
	Access10000	3.7	0.2	41.6
	Beam	1.0	0.5	11.2
	Slope	0.8	0.1	8.9
	PopDens	0.6	1.1	6.7
	TopIdx	0.5	0.4	5.6
	Hydroperid	0.9	1.9	10.1
Lower basin	Access100	7.1	1.4	35.7
	Access10000	8.5	1.8	42.7
	Beam	11.8	3.9	59.3
	Slope	1.3	0.5	6.5
	PopDens	1.7	1.2	8.5
	TopIdx	0.3	0.3	1.5
	Hydroperid	2.0	1.7	10.1

**Table 3:** Results from generalised additive models. GAM models including the most relevant variables as defined by partial deviance.

Region	Smooth terms	edf	Ref.df	Chi.sq	p-value
Upper basin	Access100	2.903	2.993	165.24	< 2 x 10^−16^***
	Access10000	2.904	2.993	39.64	2.53 x 10^−8^***
	Slope	1.358	1.615	183.09	< 2 x 10^−16^***
	PopDens	2.659	2.923	62.34	1.12 x 10^−12^***
Middle basin	Access100	2.752	2.958	93.97	< 2 x 10^−16^***
	PopDens	1.000	1.001	19.82	8.53 x 10^−6^***
	Hydroperiod	2.944	2.997	33.89	1.82 x 10^−7^***
Lower basin	Access100	1.811	2.222	32.73	3.57 x 10^−7^***
	Access10000	2.860	2.984	35.89	1.07 x 10^−7^***
	Beam	2.861	2.985	73.66	7.59 x 10^−16^***
	PopDens	1.840	2.250	27.81	1.81 x 10^−6^***
	Hydroperiod	2.076	2.434	36.95	5.15 x 10^−8^***

For the middle basin, local and regional relative accessibility were found to be the variables most strongly associated with the probability of deforestation, but only when taken individually (Table 2). Local relative accessibility, population density and hydroperiod were the most important variables in the multivariate model. The other variables explain a negligible part of the deviance in the complete model. A model which includes local relative accessibility, population density and hydroperiod (Table 3) explained the major part (7.7%) of the total deviance explained by the GAM model that includes all variables (8.9%).

Finally, for the lower basin, accessibility, hydroperiod and direct beam radiation were the variables most strongly associated with the probability of deforestation (Table 2). These variables explained the greatest proportion of the total deviance as well as a relatively high proportion of the deviance in the presence of other variables. Population density becomes an important variable when taken within a multivariate model. For this region in particular, local relative accessibility was found to be highly correlated with population density and population density explained more of the deviance than local relative accessibility when taken within a multivariate model due to predictable colinearity. A model which includes local relative accessibility, accessibility to regional markets, direct beam radiation, population density and hydroperiod (Table 3) explained the major part (19.1%) of the total deviance explained by the GAM model that includes all variables (19.9%).

Direct beam radiation was calculated using slope as an input. Slope therefore was expected to explain more of the deviance than any single derived variable taken individually due to predictable colinearity. However, for the lower basin, direct beam radiation explained more of the total deviance than slope. This suggests that direct beam radiation may be associated with some driving factor not captured by slope. This may possibly be interpreted in terms of fire frequency and intensity.

**Fig 2:**
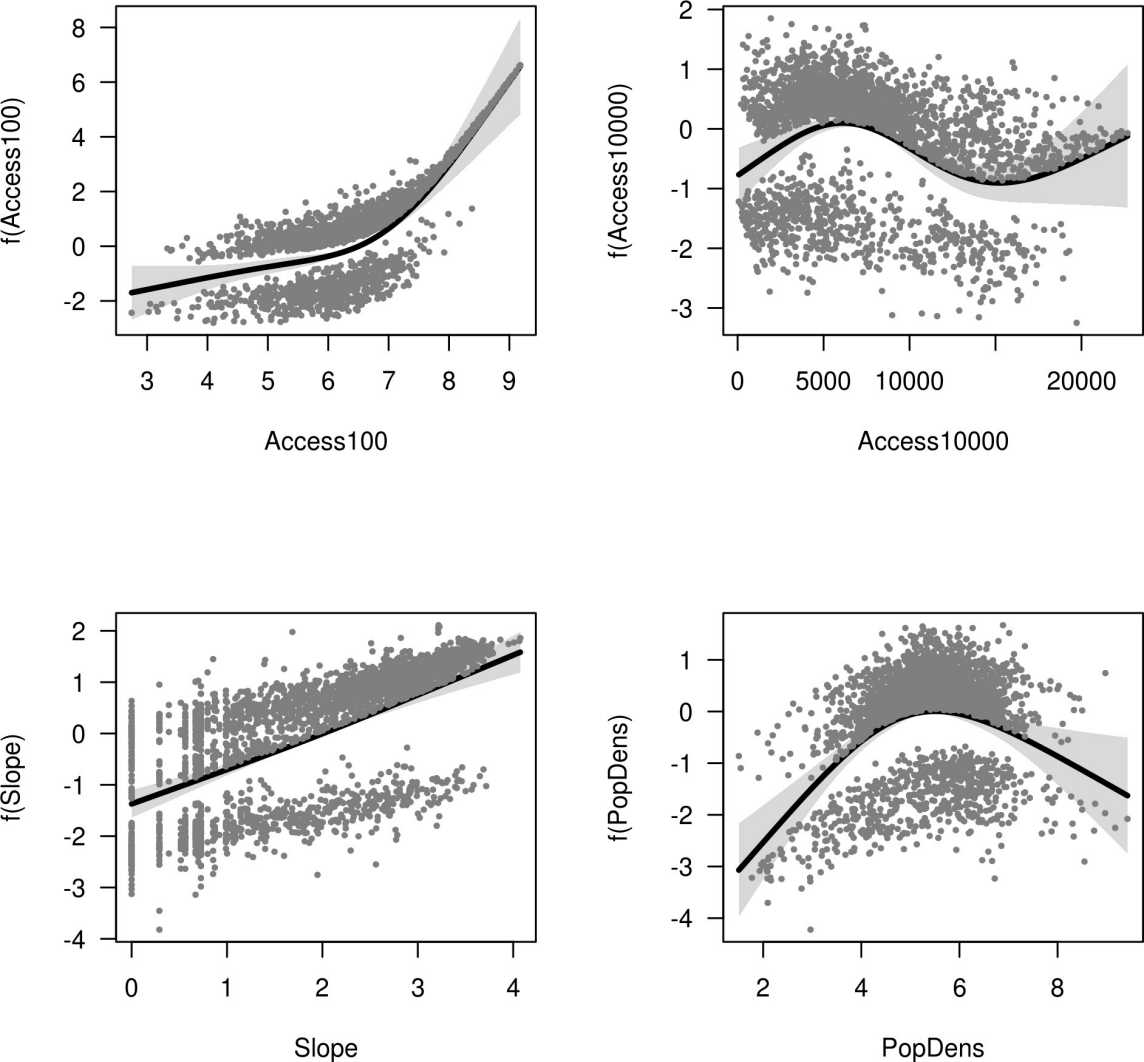
Response of vegetation cover in the upper basin. Response of vegetation cover in the upper basin to each term in a GAM model including local relative accessibility (Access100), accessibility to regional markets (Access10000), slope, and population density (PopDens). The response is on the scale of the link function. Bands show two standard errors around the response.

**Fig 3:**
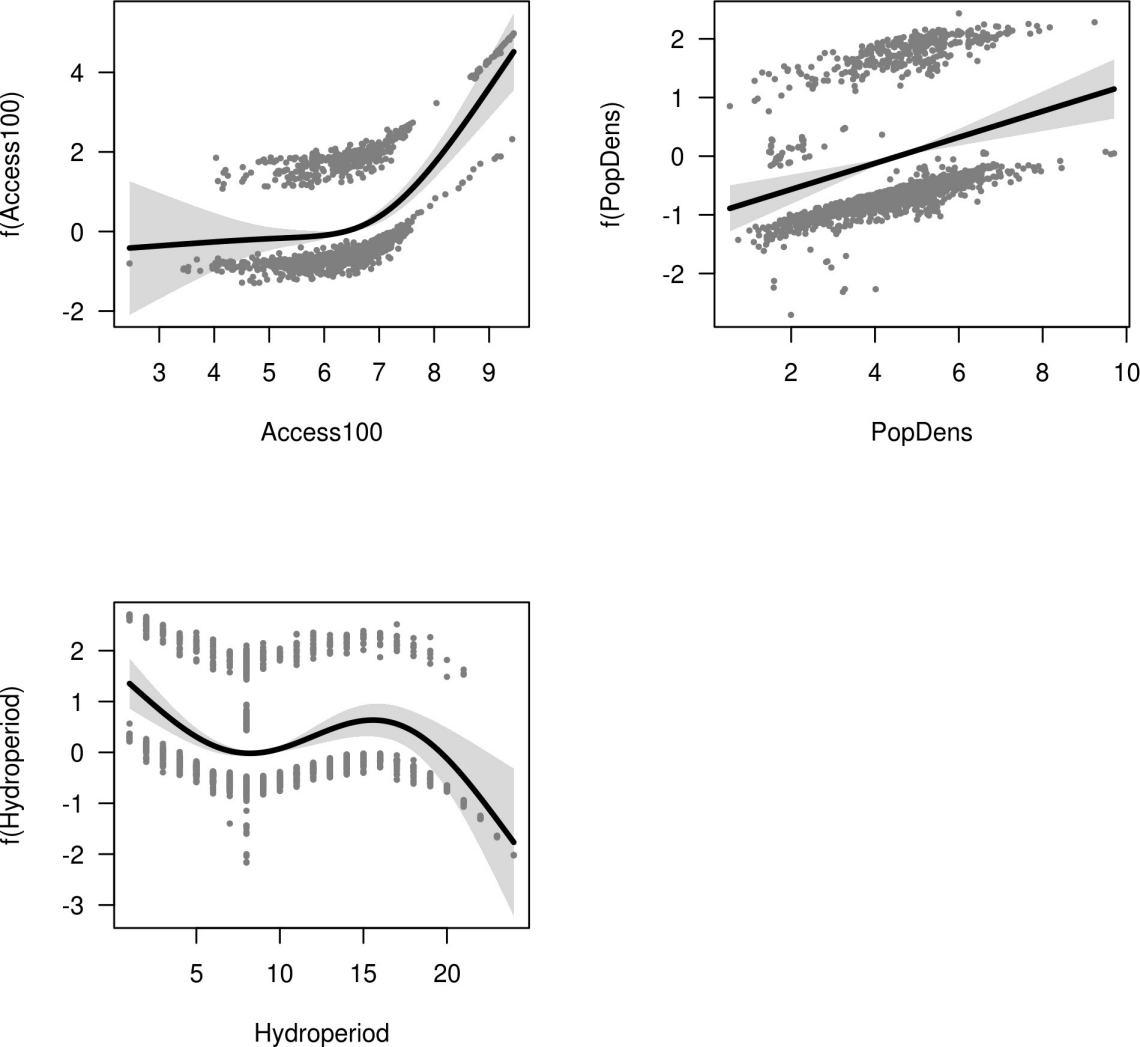
Response of vegetation cover in the middle basin. Response of vegetation cover in the middle basin to each term in a GAM model including local relative accessibility (Access100), population density (PopDens), and hydroperiod. The response is on the scale of the link function. Bands show two standard errors around the response.

**Fig 4:**
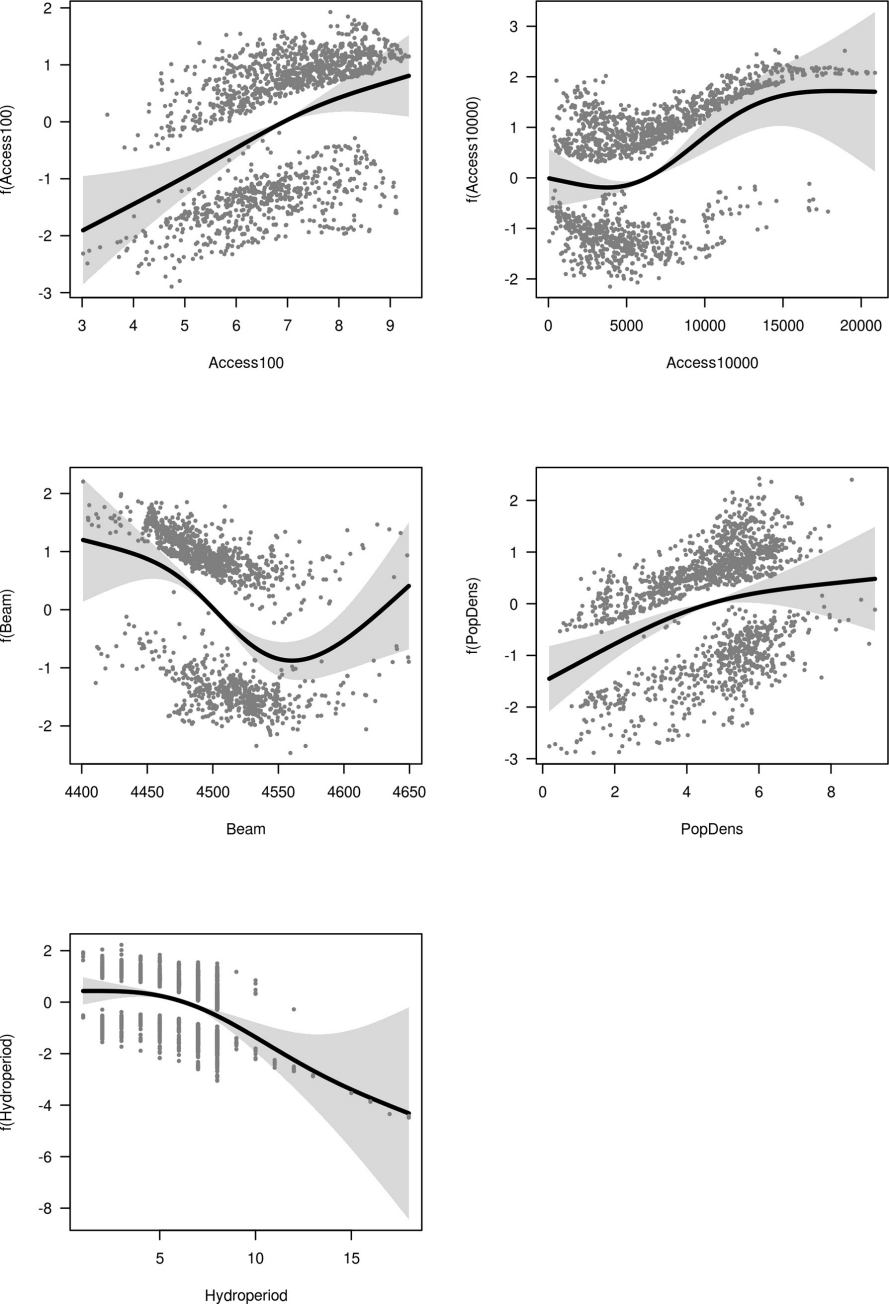
Response of vegetation cover in the lower basin. Response of vegetation cover in the lower basin to each term in a GAM model including local relative accessibility (Access100), accessibility to regional markets (Access10000), direct beam radiation (Beam), population density (PopDens), and hydroperiod. The response is on the scale of the link function. Bands show two standard errors around the response.

**Fig 5:**
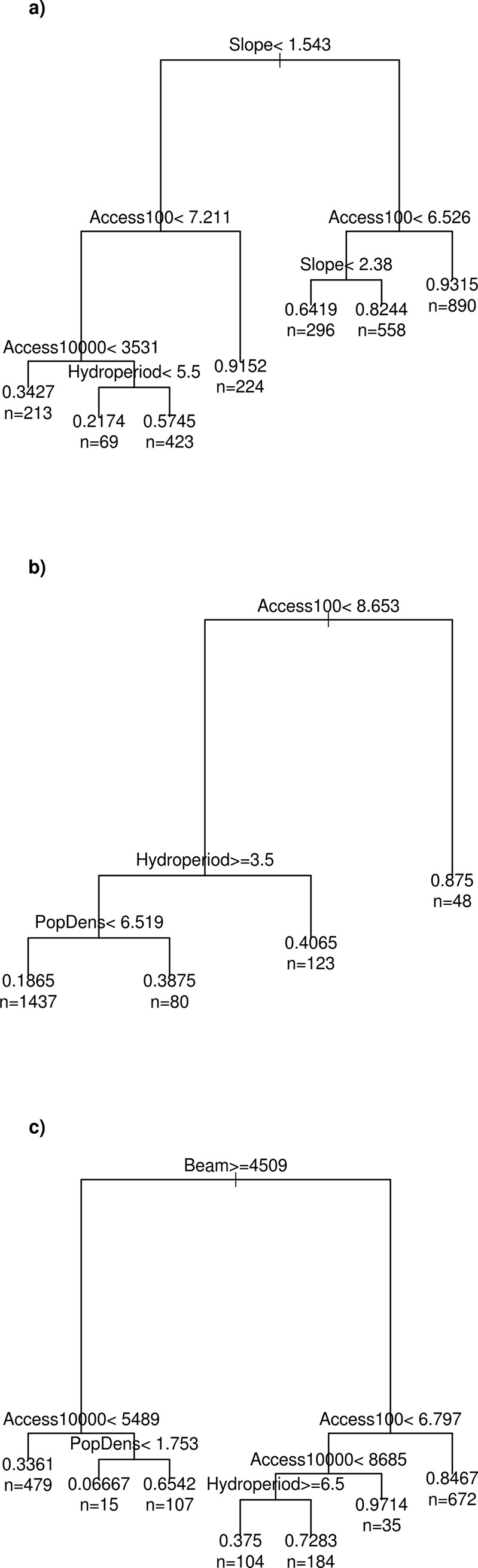
Results from recursive partitioning models. Recursive partitioning decision tree based on all predictor variables: a) upper basin; b) middle basin; and c) lower basin. The probability that a given pixel is forested can be found as a series of binary decisions. The values used are relative indices. Direct beam radiation remains an important factor in addition to slope per se as is accessibility.

Figs 2–4 show the response of vegetation cover to each term in the GAM models for the upper, middle and lower basin, respectively. The importance of the variables representing accessibility was confirmed by the recursive partitioning models (Fig 5), and there is clear evidence that direct beam insolation has an effect that is independent of slope. The recursive partitioning models were built using all the variables.

## Discussion

Several physical and socio-economic conditions were found to be important factors in explaining deforestation in the Usumacinta River Basin. The adopted methodology in this study was useful for providing insight into the comparative importance of these drivers in different parts of the region. The following discussion provides contextual explanations for the pattern of deforestation. A series of factors are used to describe where the forest is located in addition to factors influencing the decision to deforest.

### Physical factors

For the upper basin, slope was an important factor in explaining deforestation. The results suggest that the probability of a pixel to remain forested decreases with decreasing slope. Areas of gentle slopes may be preferred for agricultural and cattle raising activities, owing to potentially higher land preparation and management costs in rugged terrain. In addition, off road cost of access to available land, which is modulated by terrain and topography, may be a critical factor; therefore, areas of steep slope are not likely to be deforested. Previous research in other tropical forest regions embedded within major mountain systems in Mexico (e.g. [48–50]), show that the most negatively affected areas by permanent deforestation are those with gentle slopes.

The upper basin of the Usumacinta River includes a mixture of federal, private and communal property regimes, with *ejido* lands constituting the majority (>50 per cent) of its area [51]. [51] used a typology based on policy-relevant farmer characteristics (land tenure, farm size, source of income, farming system) to differentiate between farmers (traditional vs. cattle ranching) with different motivations that determine how management affects landscape configuration in this region. Major deforestation in the upper basin, coincided spatially with low-sloped areas, and according to [51] these areas are dominated by large-scale commercial farms that specialize in livestock production, as well as with large units with low-sloped high quality land within *ejidos*, which are managed by traditional peasant farmers involved in a mix of subsistence and market production. Policies in the livestock sector affect deforestation by influencing the incentives to convert forest land for this economic activity, rather than maintain the forest for other uses. The comparative returns gained from converting forests to livestock production are a major factor determining deforestation [52]. Land intensification by peasant farmers decreased as the landscape became increasingly rugged. In these areas traditional farmers carry out other economic activities. They have areas devoted to coffee, xate, and fruit production for self-consumption and sale [51]. Forest resources in the region are used basically for self-individual consumption and are not exploited commercially due to past overexploitation of primary forests, which resulted in a low density of species with commercial value [51].

Agricultural activities may depend not only on the physical characteristics of the terrain but also on the natural cycles of water excesses. Particularly, in the middle (floodplain) and lower (coastal plain) basins, the hydroperiod was found to be an important factor associated with the probability of deforestation. The probability of agricultural activities decreases as water levels are nearer the surface. Water levels near the surface maybe associated with the probability of accumulating water and unfavourable moisture conditions for cropping.

Finally, the analysis confirmed the importance of dry season insolation as an important factor in deforestation, particularly in the lower basin. The use of fire is a common feature in this region. Fire is used to burn open brushlands and wetlands, and to keep areas free of trees and improve poor quality forage for livestock grassing [53–54]. Dry weather makes it easier to burn wetlands and marshes in the winter. Areas that receive more direct beam insolation in the dry season tend to be warmer and drier and, therefore, fuel may dry out quickly leading to severe fires. At the same time, deforested areas that receive more direct beam insolation in the dry season are more likely to be permanently cleared.

### Socio-economic factors

The effect of accessibility was clearly shown by the analysis. Areas that are highly accessible tend to be more productive. Local relative accessibility (i.e. cost of access to available land from rural settlements) and regional relative accessibility or accessibility to regional markets (i.e. accessibility to urban centres), lead to different inferences because the models have different interpretation.

Urban population requirements associated with high demand for agricultural products may encourage marketable agricultural production [55]. In turn, accessibility to regional markets associated with the local performance of the economy may influence the decision regarding agricultural land use. Particularly, in the lower basin, accessibility to urban centres was shown to be much more important than local accessibility. Major urban centres in the study region are concentrated in the lower basin. The current pattern of usage in most of the coastal floodplain has origins both in past hydraulic plans implemented in the 1950s, 60s, and 70s, that aimed at improving land for agriculture (through wetland drainage and burning), and in sectoral policies aimed at raising production of key agricultural and livestock products and at incorporating the region into the national economy [40]. Industry-based agriculture, flourished on the better lands and larger properties [31]. In addition, a large proportion of the coastal floodplain is populated by modestly subsidized small farmers using low-external-input technologies which provide cheap staple food for the urban market of Villahermosa (the largest urban centre in the study region) and other urban centres located in the coastal floodplain [56–57]. The importance of regional relative accessibility in the lower basin may therefore reflect the influence of factors such as market access, infrastructure, and transportation costs. On the other hand, part of the coastal floodplain is characterised by being environmentally restrictive for agricultural use, with flooding imposing great difficulties for accessing most of the land. This explains the strong effect of local relative accessibility.

Local relative accessibility was also an important driver in the middle and upper basins. Small farmers in the upper basin live in a highly scattered settlement pattern and are predominantly subsistence farmers. In addition, commercialization of products is constraint by the unfavourable state of rural infrastructure needed to facilitate the distribution of products. Therefore, the selection of areas for agricultural expansion may be influenced mainly by cost of access to available land. Moreover, local relative accessibility in the upper basin is thought to be associated with slope which was used in its calculation.

The association between population density and the proportion of forested pixels does not provided support to the conclusion that population density play a direct role in deforestation. Other research in the region [58], has reported that human population density appears to play a limited role in explaining the variation in deforestation. In the present research, the association between population density and the proportion of forested pixels retains strong value for providing contextual explanations for deforestation based on socio-economic and political factors. The likelihood of a pixel to remain forested was shown to increase with increasing population density. Population density may be associated with some other driving factor, and this may possibly be interpreted in terms of the role of land tenure in shaping the decision to deforest. Patterns of land tenure were included as solid barriers for the calculation of total population at the pixel level. The most profitable land throughout the study region is mostly retained into large private properties within which extensive agricultural and cattle raising activities are developed by capitalised landowners. These landowners reside mainly in urban centres and these profitable zones are comparatively sparsely populated. Thus population density has not played a direct role in deforestation over most of the study area. In contrast, *ejidos* properties with resident rural communities and the highest levels of population density are found at the most rugged and inaccessible areas (upper basin), or at environmentally restrictive areas (lower and middle basins), that are residual from intensive uses. These rural communities are characterized by a strong dependency on forest resources and a limited economic development that eventually dwindle the forest frontier. The agricultural production of this zone is used for subsistence, and remaining forest is retained in the most remote and inaccessible areas and it is managed for its utility as a source of fuelwood and timber. This fact explains, to a large extent, why the likelihood of a pixel to remain forested increases with increasing population density. Other authors [59–60] have shown that smallholders in Calakmul and other areas surrounding the Usumacinta River Basin typically maintain significant areas in mature forests and secondary forest succession. In summary, the likelihood of a pixel to remain forested decreases with decreasing population density as the pattern of land tenure becomes dominated by large private properties. Also, government investment, specifically in agricultural intensification and forest and soil conservation in *ejidos* properties, appears to reduce deforestation [51]. Finally, the development of ecotourism projects and other conservation initiatives by many rural communities generate funds whose investment in public goods increases the value of standing forest [13,51,61].

### Policy implications

The results of this study suggest that not all segments of the rural population are equally important drivers of deforestation. This finding is in accordance with the results from other research (e.g. [51,62–63]). Large-scale ranchers, rather than small-scale farmers, appear to be responsible for most of the loss of native vegetation in the Usumacinta River Basin. In contrast, forest exploitation by people who live in resource-dependent rural communities is often relatively intensive. In other study, [64] showed that rates of forest cover loss in central Yucatan Peninsula during the period 2005–2015 were significantly higher in private and federal property compared to forests in *ejidos* (communal property). Institutional conservation and development policies are therefore needed that make sense in the particular context of these two different segments of the rural population. Other authors [13,51,59] have pointed out that in order to address policy options for fostering conservation and sustainable development in Mexico, it is necessary to understand the heterogeneity of land tenure systems and the specificity of landowners decision-making process.

We believe that policies directed at promoting the diversified and sustainable use of forests as well as alternative agricultural techniques that maintain and/or increase the productivity of cultivated land, would be more effective in reducing the likelihood of deforestation in *ejidos* properties while maximizing rural livelihoods, than policies focused on direct actions for avoiding deforestation or the prohibition of certain traditional land and forest uses. Other research supports this belief [51]. Future actions should include, for example, the development of fuelwood plantations, the creation of ecotourism developments, and the management of forests for sustainable commercial logging. Governmental financial resources can also benefit collaboration by facilitating research, providing technical assistance, covering operating expenses and creating plans. On the other hand, those *ejidos* in which physical and economic conditions favour deforestation should be required to examine and redefine their rules in order to maintain or increase the proportion of forest area. Strategies focused on reducing deforestation and raising the profitability of the forest relative to agriculture, such as payments for environmental services, should be given to communities with forests at higher risk of forest loss, namely large *ejidos* with low-sloped land of high quality. In contrast, the economic valuation of the forest coupled with the promotion of plans for the restoration of degraded pastures and the regeneration of woody vegetation, should be helpful in raising the awareness of private landowners and small-scale cattle farmers about the importance of these forests. Areas undergoing regeneration are likely to sequester more carbon and provide other ecological services such as soil and water conservation.

To date, almost all of the strategies focused on reducing deforestation are directed at small-scale farmers. There are several reasons for a prioritization on this segment of the rural population: (1) small-scale farmers are attentive to financial concerns, (2) traditional agricultural technology provides advantageous opportunities for developing processes for rehabilitating and restoring ecosystem functions and conserving biodiversity [60], (3) poverty alleviation, and (4) conveying robustness and resilience to local livelihoods. Nevertheless, despite clear reasons for a focus on small-scale farmers, strong action is also required to take on the private sector.

Within the protected areas of Pantanos de Centla and Laguna de Términos (and along the lower basin in general), the number of cattle grazing extensively within their buffer zones has increased rapidly in the last two decades. Agrarian extension has led not only to direct, deliberate removal of the native vegetation in order to provide pasture, but also to an increase in fire severity [56,65]. Fire plays a key role in habitat degradation [66]. Protected areas within the study region are partially successful in mitigating the negative impacts of fire [67–68]. The loss of vegetation leads to both chronic and acute soil erosion. Trampling and browsing by cattle prevents tree seedlings establishing. The result is generally agreed by both residents and researchers in the region to be causing degradation in the long term productivity [65]. The results of this study provide additional information that may be useful in understanding how the cumulative effects of peasants land use practices lead to chronic deforestation of wetlands and shrublands in the lower basin. The effect of winter insolation was found to have many applied implications for management of these zones. It appears that direct beam insolation is associated with deforestation on two counts. 1) Fuel dries out quickly leading to severe fires. 2) Regeneration is prevented by hydric stress experienced by juvenile trees and shrubs. Therefore, less remote areas may be undergoing a form of chronic deforestation through the cumulative effects of fires, uncontrolled browsing and poor regeneration. It does indeed seem to be that permanent deforestation is arising from poor regeneration. This information can supports further research in the area and suggests more sustainable management practices.

Finally, our findings indicate that population density does not appear to play a determining role in deforestation and, as it has been stated in other studies [58], it may be unwarranted to use human demographic information to predict deforestation and to imply causality.

## Conclusions

Our research was conducted in response to the lack of studies focused on determining the causes of environmental degradation and deforestation in Mexico's tropical regions. We used a methodological approach specifically designed to avoid a set of weaknesses that are common to traditional statistical methods and that arise when observational data on spatially explicit phenomena have not arisen as a result of planned experimentation. The methodology probes to be successful for interpreting the relative importance of a series of physical and socio-economic factors responsible for the pattern of historical deforestation and for providing contextual explanations for this pattern. It resulted in an informative methodological approach, thus allowing the construction of a comprehensive understanding of the causes of deforestation that can supports research and inform decision making.

The proposed approach, however, could not completely overcome some of the problems associated with spatially-explicit modelling, which is evident given the amount of variance that remains to be explained, possibly due not only to the underlying unexplainability of human behaviour but also as a result of exclusion of variables that are difficult to spatialise. Many processes may in fact best be observed at ground level through a more anthropological approach, and these types of data often relate to variables that cannot be mapped onto space. The ideal would probably be an approach in which this kind of spatial analysis is enriched with such data.
