# Correction: Drivers of deforestation in the basin of the Usumacinta River: Inference on process from pattern analysis using generalised additive models

**DOI:** 10.1371/journal.pone.0228328

**Published:** 2020-01-23

**Authors:** Raúl Abel Vaca, Duncan John Golicher, Rocío Rodiles-Hernández, Miguel Ángel Castillo-Santiago, Marylin Bejarano, Darío Alejandro Navarrete-Gutiérrez

The affiliation for the third author is incorrect. The correct affiliation is not indicated. Rocio Rodiles-Hernández is not affiliated with #1 but with Departamento de Conservación de la Biodiversidad, El Colegio de La Frontera Sur, San Cristóbal de Las Casas, Chiapas.
